# Supplementary material for: Epigallocatechin gallate from green tea effectively blocks infection of SARS-CoV-2 and new variants by inhibiting spike binding to ACE2 receptor
Source: Cell Biosci. 2021 Aug 30;11:168. doi: 10.1186/s13578-021-00680-8 (PMC8404181; doi:10.1186/s13578-021-00680-8)
Supplement: Supplementary file 1 — Additional file 1: Figure S1. Generation of VSVΔG-based pseudovirus bearing SARS-CoV-2 S protein (SARS-CoV-2 pseudovirus). Figure S2. Verification of the incorporated SARS-CoV-2 spike in the pseudovirus. Figure S3. Green tea beverage (GTB) and the catechins have little cytotoxicity on the cells. Figure S4. EGCG has no effect on luciferase activity and luciferase gene expression. [file 13578_2021_680_MOESM1_ESM.pdf]

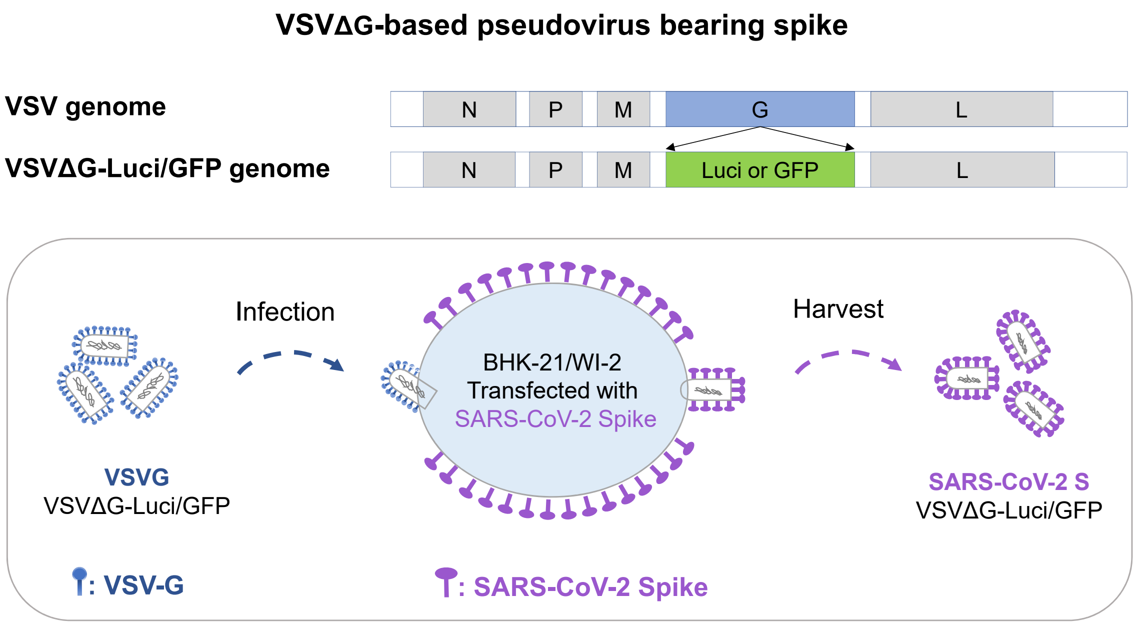

Figure S1. Generation of VSVΔG-based pseudovirus bearing SARS-CoV-2 S protein (SARS-CoV-2 pseudovirus). BHK-21/WI-2 cells were transfected with pCAG-SARS-CoV-2 S plasmids 24 h prior to VSVΔG-Luci/GFP-based pseudovirus bearing VSVG (VSVG-VSVΔG-Luci/GFP) infection (MOI=4). The cells were washed with PBS for 3 times to remove unbounded VSVG-VSVΔG-Luci/GFP at 2 h postinfection, and cultured in 5% DMEM for 24 h. Virions-containing supernatant was harvested and centrifuged at 500 g for 5 min, filtered with 0.45 μm polyethersulfone membrane.

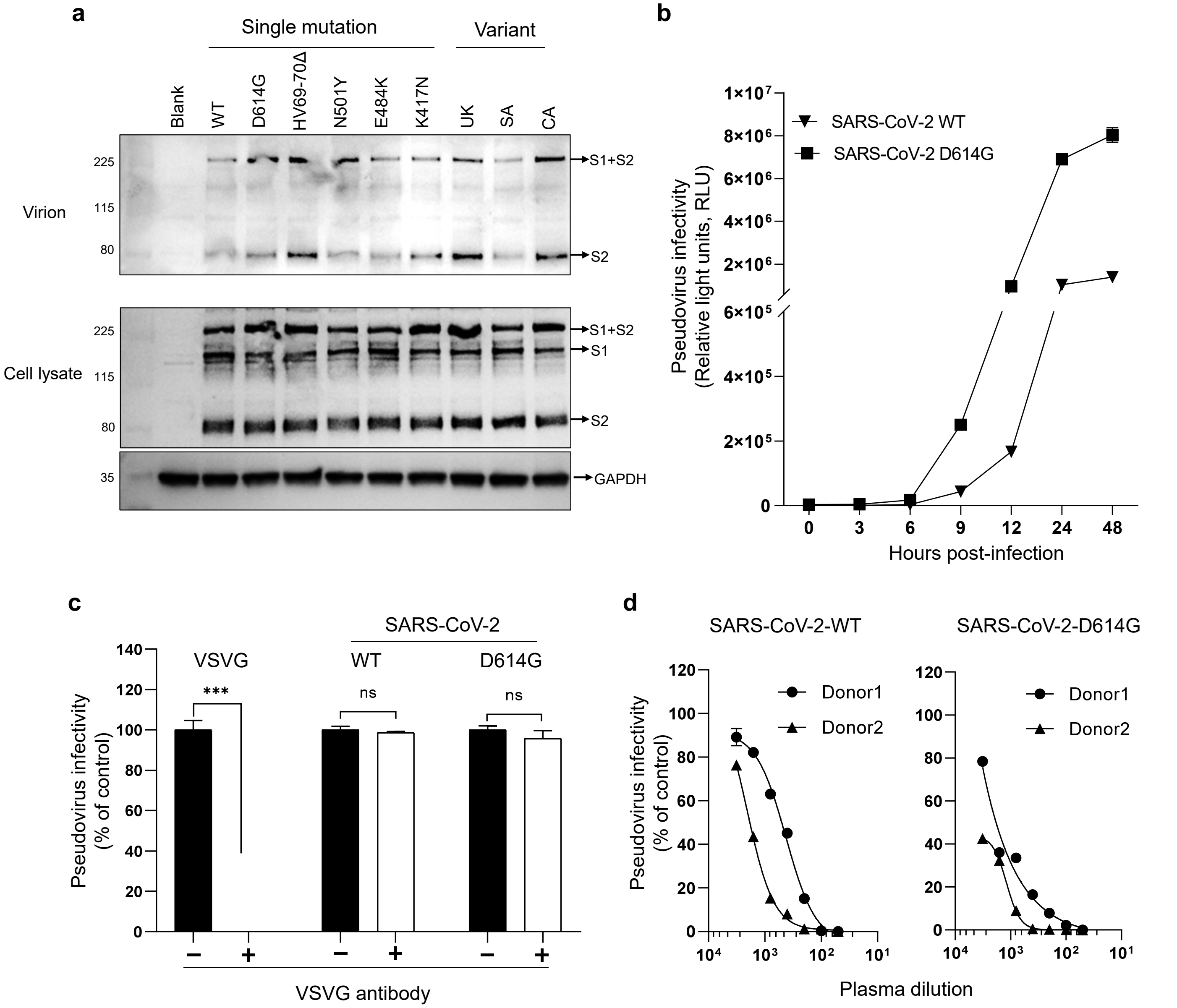

Figure S2. Verification of the incorporated SARS-CoV-2 spike in the pseudovirus. (**a**) Virion-containing supernatant and producer cells (BHK-21/WI-2) were harvested and subjected to western blot using SARS-CoV-2 convalescent plasma as the detection antibody (1:500 dilution). GAPDH was used as a loading control of western blot. (**b**) HEK239T-hACE2 cells were infected with VSVΔG-based pseudovirus bearing SARS-CoV-2 spike (SARS-CoV-2 pseudovirus) for indicated time, cells were lysed and subjected to luciferase activity detection. (**c**) The specificity of SARS-CoV-2 pseudovirus infection was tested against VSVG-specific antibody using VSVG-VSVΔG-Luci pseudovirus as the control. The data represent percentage infectivity relative to that of the untreated group. (**d**) Plasma samples from 2 convalescent patients of COVID-19 were serially diluted and incubated for 1 h at 37°C with indicated SARS-CoV-2 pseudoviruses and infectivity in HEK293T-hACE2 cells was then measured. The data represent percentage infectivity relative to that of the control (untreated). ****P*<0.001.

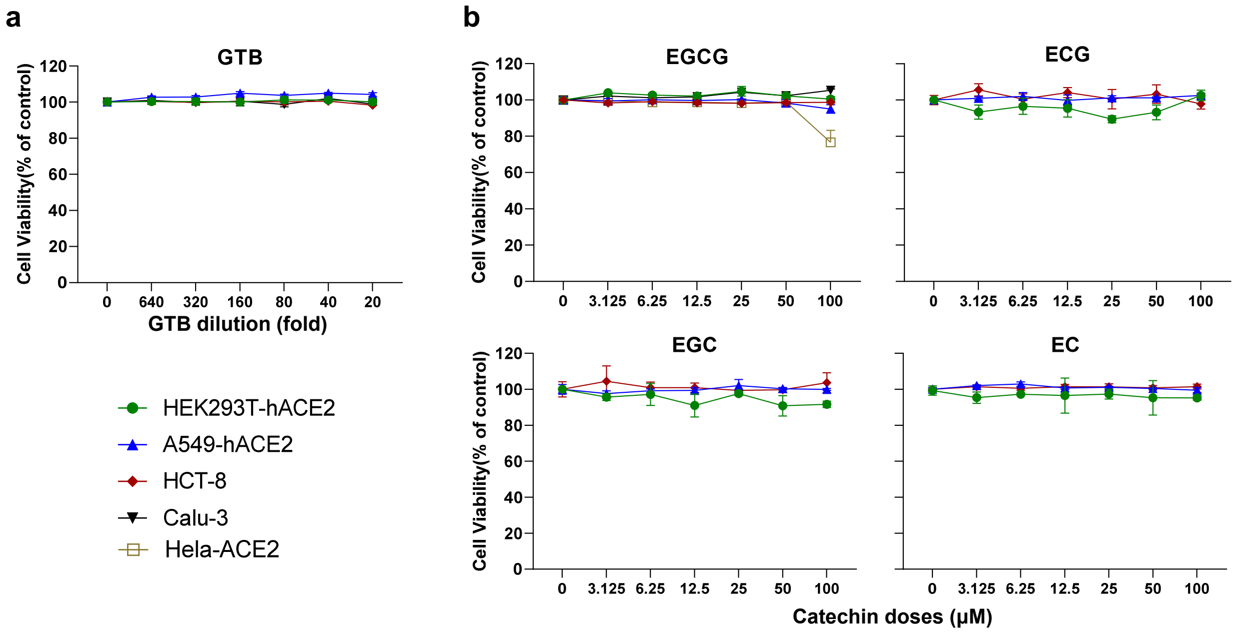

Figure S3. Green tea beverage (GTB) and the catechins have little cytotoxicity on the cells. (**a, b**) Cells (HEK293T-hACE2, A549-hACE2, HCT-8, Calu-3 and Hela-ACE2) were treated with GTB or the catechins (EGCG, ECG, EGC and EC) at the indicated doses for 72h. The cell viability was assessed by MTS assay. Data represent percentage changes of the absorbance (490 nm) relative to untreated control.

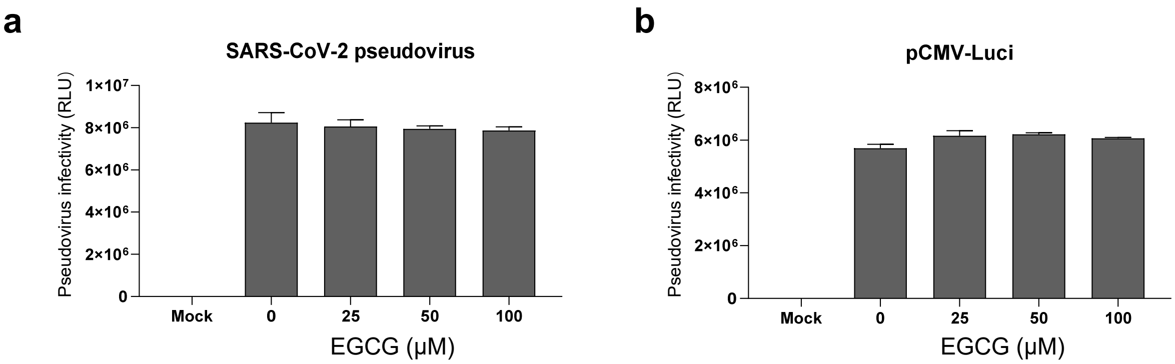

Figure S4. EGCG has no effect on luciferase activity and luciferase gene expression. (**a**) HEK293T-hACE2 cells were infected with VSVΔG-based pseudovirus bearing SARS-CoV-2 S (SARS-CoV-2 pseudovirus) for 48 h, and then lysed, different dose of EGCG was added in the cell lysate when measuring luciferase activity. (**b**) HEK293T-hACE2 cells were transfected with plasmid encoding luciferase, then treated with or without EGCG (0~100 μM) for 48 h. Cells were lysed and subjected to luciferase activity detection.
