# Supplementary material for: Epigallocatechin gallate from green tea effectively blocks infection of SARS-CoV-2 and new variants by inhibiting spike binding to ACE2 receptor
Source: Cell Biosci. 2021 Aug 30;11:168. doi: 10.1186/s13578-021-00680-8 (PMC8404181; doi:10.1186/s13578-021-00680-8)
Supplement: Supplementary file 2 — Additional file 2: Table S1. Mutagenic primers for each mutant of full-set S variants. [file 13578_2021_680_MOESM2_ESM.docx]

| **Table S1.** Mutagenic primers for each mutant of full-set S variants | | |
| --- | --- | --- |
| **Construct** | **Primer** | **Sequence (5’ to 3’)** |
| **D614G Mutation**  (using Sd18 wildtype construct as template) | Sd18-EcoRI-UTR-F | ctcatcattttggcaaagaattcattaaaggtttataccttccc |
|  | Sd18-D614G-R | cacgccctggtacagcacggccacct |
|  | Sd18-D614G-F | aggtggccgtgctgtaccagggcgtgaattgcaccgaggtgcc |
|  | Sd18-D614G-PpuMI-R | caccttgttgaacagcaggtcct |
| **UK Variant B.1.1.7**  (using D614G construct as template) | Sd18-∆H69V70-R | accgcttggtgccattggtgccgctgatggcgtggaaccaggtcac |
|  | Sd18-∆H69V70-F | agcggcaccaatggcaccaagcgg |
|  | Sd18-∆Y144-R | ccaggacttattgttcttgtggtacacgcccaggaatggatcattg |
|  | Sd18-∆Y144-F | taccacaagaacaataagtcctgg |
|  | Sd18-N501Y-R | tgtatggctggtatcccactccgtaggttggctggaagccgtagct |
|  | Sd18-N501Y-F | acggagtgggataccagccataca |
|  | Sd18-A570D-R | cgcacggcgtcggtggtatcatcgatgtcccttccgaactgctg |
|  | Sd18-A570D-F | tgataccaccgacgccgtgcg |
|  | Sd18-P681H-R | ggctggccacgctgcgagccctcctgtgggagttggtctgggtctggtag |
|  | Sd18-P681H-F | aggagggctcgcagcgtggccagcc |
|  | Sd18-T716I-R | ggtggtcacggagatggtgaagttgattgggatggcgatggaattgttgc |
|  | Sd18-T716I-F | caacttcaccatctccgtgaccacc |
|  | Sd18-S982A-R | cagcctccaccttgtccaggcgggccaggatatcattcagcacgctgg |
|  | Sd18-S982A-F | ccgcctggacaaggtggaggctg |
|  | Sd18-D1118H-R | cagttgccgctcacgaaggtattgtgggtggtgatgatctggggctcgtag |
|  | Sd18-D1118H-F | acaataccttcgtgagcggcaactg |
|  | Sd18-Not-R | ctgataggcagcctgcacctgaggagttacttgcagcagctgccgcaggagcagcagc |
| **South African Variant B.1.351**  (using D614G construct as template) | Sd18-L18F-R | caggaggcagctgggttctagtagtgaagttcacgcactgagacgagaccag |
|  | Sd18-L18F-F | actactagaacccagctgcctcctg |
|  | Sd18-D80A-R | catcgttgaatggcagcacgggattggcgaaccgcttggtgccattggtgcc |
|  | Sd18-D80A-F | aatcccgtgctgccattcaacgatg |
|  | Sd18-D215G-R | cagggcgctgaagccctgtggcaggccgcgcaccaggttgatgggggtg |
|  | Sd18-D215G-F | tgccacagggcttcagcgccctg |
|  | Sd18-∆LAL-R246I-R | agctgtcgcctggggtcaggtagctgatgtgcagggtctggaacctggtgatgttg |
|  | Sd18-∆LAL-R246I-F | cagctacctgaccccaggcgacag |
|  | Sd18-K417N-R | agcttgtaattgtagtcagcgatattgccggtctgtcctggagcga |
|  | Sd18-K417N-F | cagatcgctgactacaattacaagct |
|  | Sd18-E484K-R | ggaagtagcagttgaagcccttcactccattgcatggggtgcttc |
|  | Sd18-E484K-F | aagggcttcaactgctacttcc |
|  | Sd18-N501Y-R | tgtatggctggtatcccactccgtaggttggctggaagccgtagct |
|  | Sd18-N501Y-F | acggagtgggataccagccataca |
|  | Sd18-A701V-R | gttgctgtaggccacgctattctcgacgcccagggacatggtgtaggcgatg |
|  | Sd18-A701V-F | cgagaatagcgtggcctacagcaac |
| **US California Variant B.1.429**  (using D614G construct as template) | Sd18-S13I-R | ctagtagtcaggttcacgcactgaatcgagaccagaggcagcaggaccagg |
|  | Sd18-S13I-F | tcagtgcgtgaacctgactactag |
|  | Sd18-W152C-R | gtacacgcggaactcgctctccatacaggacttattgttcttgtggtag |
|  | Sd18-W152C-F | atggagagcgagttccgcgtgtac |
|  | Sd18-L452R-R | gattggacttgcggaacaggcggtaccggtaattgtagttgccgcccactttg |
|  | Sd18-L452R-F | gtaccgcctgttccgcaagtccaatc |
